# Association between supportive supervision and performance of community health workers in India: a longitudinal multi-level analysis

**DOI:** 10.1186/s12960-021-00689-7

**Published:** 2021-11-27

**Authors:** Lakshmi Gopalakrishnan, Nadia Diamond-Smith, Rasmi Avula, Purnima Menon, Lia Fernald, Dilys Walker, Sumeet Patil

**Affiliations:** 1 University of California Berkeley, Berkeley, USA; 2 University of California San Francisco, San Francisco, USA; 3 International Food Policy Research Institute, Washington, DC USA; 4 NEERMAN, Center for Causal Research and Impact Evaluation, Mumbai, India

**Keywords:** Anganwadi worker, CHWs, ICDS, Integrated Child Development Services, Supervision, Nutrition, Madhya Pradesh, Bihar, Supervisor, Health system

## Abstract

**Introduction:**

Community health workers (CHWs) deliver services at-scale to reduce maternal and child undernutrition, but often face inadequate support from the health system to perform their job well. Supportive supervision is a promising intervention that strengthens the health system and can enable CHWs to offer quality services.

**Objectives:**

We examined if greater intensity of supportive supervision as defined by monitoring visits to Anganwadi Centre, CHW-supervisor meetings, and training provided by supervisors to CHWs in the context of Integrated Child Services Development (ICDS), a national nutrition program in India, is associated with higher performance of CHWs. Per program guidelines, we develop the performance of CHWs measure by using an additive score of nutrition services delivered by CHWs. We also tested to see if supportive supervision is indirectly associated with CHW performance through CHW knowledge.

**Methods:**

We used longitudinal survey data of CHWs from an impact evaluation of an at-scale technology intervention in Madhya Pradesh and Bihar. Since the inception of ICDS, CHWs have received supportive supervision from their supervisors to provide services in the communities they serve. Mixed-effects logistic regression models were used to test if higher intensity supportive supervision was associated with improved CHW performance. The model included district fixed effects and random intercepts for the sectors to which supervisors belong.

**Results:**

Among 809 CHWs, the baseline proportion of better performers was 45%. Compared to CHWs who received lower intensity of supportive supervision, CHWs who received greater intensity of supportive supervision had 70% higher odds (AOR 1.70, 95% CI 1.16, 2.49) of better performance after controlling for their baseline performance, CHW characteristics such as age, education, experience, caste, timely payment of salaries, Anganwadi Centre facility index, motivation, and population served in their catchment area. A test of mediation indicated that supportive supervision is associated indirectly with CHW performance through improvement in CHW knowledge.

**Conclusion:**

Higher intensity of supportive supervision is associated with improved CHW performance directly and through knowledge of CHWs. Leveraging institutional mechanisms such as supportive supervision could be important in improving service delivery to reach beneficiaries and potentially better infant and young child feeding practices and nutritional outcomes.

*Trial registration :* Trial registration number: https://doi.org/10.1186/ISRCTN83902145

**Supplementary Information:**

The online version contains supplementary material available at 10.1186/s12960-021-00689-7.

## Background

Community health workers (CHWs) are integral to healthcare delivery in rural and hard-to-reach areas of low- and middle-income countries (LMICs) [1]. The term “Community health workers” covers a broad category of lay and educated, formal and informal, paid and unpaid, health workers. Their role typically involves educating community members about health risks, promoting health behaviors, and referring community members to formal health system services [2]. Task shifting from specialist health workers to generalist CHWs is commonplace in LMICs, especially when faced with severe shortages and inequitable distribution of skilled personnel [3]. Consequently, CHW-driven programs are a feature of many national health systems.

Previous evidence has shown that CHW programs have successfully delivered a range of services, including preventive, promotive, and curative care across many health and nutrition outcomes in low-resource settings [4–7]. CHWs have improved infant and child feeding practices and supported children with undernutrition and micronutrient deficiencies [1]. A large-scale CHW program in Mozambique showed a one-third reduction in the prevalence of childhood underweight status [8]. A considerable amount of research synthesized in various systematic reviews highlights that CHWs programs have successfully improved breastfeeding practices and immunization uptake, reduced pneumonia, diarrhea, and delayed bottle feeding, and promoted essential newborn care [4, 9, 10]. A slightly dated review of CHWs from India found robust evidence of the positive role CHWs play in expanding vaccination coverage by targeting hard-to-reach households, timely tracking of children, and sensitizing rural communities about vaccination services [11].

Since the Alma-Ata Declaration of 1978 identified primary healthcare as a critical mechanism to achieve Health for All, there has been a growing recognition that CHWs, often selected and supported by communities, cannot be left to serve on their own [12]. Several challenges plague the CHW programs, including less supervision, limited planning, inadequate logistical support, untimely payments, overlapping roles with other CHWs but poor convergence, fragmented training, weak linkages to health systems, low trust among community members, and under-recognition of CHWs’ contributions [7, 13, 14].

Supportive supervision to CHWs is acknowledged as an essential lever to ensure that CHWs perform well, remain motivated, and have well-defined roles within the health system [14–16]. Supervisors form the link between CHWs and health systems [17–20]. Recent reviews on CHW programming have endorsed the role supportive supervision could play in improving the knowledge, productivity, and performance of CHWs [2, 7, 14]. Unlike traditional supervision that includes oversight and control of CHWs by senior health system staff, supportive supervision includes elements of record reviews, observations, performance monitoring, constructive feedback, problem-solving, and training to support the CHWs in delivering services [19].

There is some evidence to suggest that supportive supervision can help CHWs in improving their performance and motivation [7, 20, 21]. For example, a time-use study in Ghana reported that health workers who received supportive supervision spent more time providing direct patient care than workers who did not receive supportive supervision (OR 2.37, *p* < 0.01) [22]. In Nicaragua, Perez and colleagues found an improvement in CHW quality of care for newborns and early initiation of breastfeeding through a pilot intervention package involving neonatal care training and supportive supervision [23]. A randomized controlled study in Mali found that dedicated monthly supervision with customized feedback via dashboards to each CHW improved the number of home visits delivered by CHWs [24]. India-specific evidence on gains made from supportive supervision is limited to one quasi-experimental study in Odisha. This intervention-based Odisha study found that supervisors who were provided training on supervision techniques were better equipped to supervise CHWs, which further improved knowledge and service delivery of CHWs providing immunization services [25, 26].

However, the evidence on the effectiveness of supervision remains inconsistent. A mixed-methods study examining the effect of a group supervision intervention involving training and mentorship of supervisors in Ethiopia, Kenya, Malawi, and Mozambique did not find statistically significant improvement in CHW motivation and performance. However, qualitative evidence suggested that supervision improved CHW motivation [27]. A cluster-controlled trial of facility health workers in Mozambique found no statistically significant impact of supportive supervision on job satisfaction, emotional exhaustion, and work engagement. But the qualitative interviews revealed that health workers perceived supervision to increase their motivation levels [28].

## Context of India’s flagship nutrition program, the Integrated Child Development Services (ICDS)

The Integrated Child Development Services (ICDS) is one of India’s flagship nutritional programs focused on providing nutrition and health-related services to pregnant and lactating women and children under the age of six. The program is delivered through a network of 1.4 million Anganwadi workers (*henceforth referred to as CHWs*) based in the Anganwadi Centers (AWCs), the early childhood development and feeding centers.

Specifically, these CHWs support the nutrition program by providing: (i) supplementary food including hot cooked meals and take-home rations; (ii) home visits to educate pregnant and lactating women on pregnancy care, and infant and young child feeding practices; (iii) growth monitoring activities of children as appropriate for each age group, (iv) pre-school education to 3–6 year old children, and (v) monthly fixed-day event—village health and nutrition days (VHND) for immunization and other health-related services [29]. A typical CHW is a part-time female worker who receives an average monthly fixed honorarium of about USD 60 (INR 4500), though the honorarium varies by each state. They provide services to a catchment area covering approximately 800–1000 children below six and pregnant and lactating mothers [29–31]. The ICDS program also has a dedicated cadre of supervisors who manage a cluster of 20–25 CHWs [25]. These supervisors conduct monthly monitoring visits to the Anganwadi Centers (AWCs) to identify gaps in service delivery and to support the CHWs in delivering their services, hold monthly meetings with CHWs to review progress and delivery-related challenges, and train CHWs on topics related to nutrition, pregnancy care, infant feeding, etc.

Several challenges impact nutrition service delivery in India, including incomplete record-keeping, inadequate monitoring, lack of timely service delivery, ill-equipped centers, insufficient training and supervision [32–36]. For example, national survey data suggest that only 59% of mothers reported receiving supplementary nutrition, growth monitoring, and pre-school education services from CHWs for children below six [37]. Supportive supervision could be one of the supply-side strategies to strengthen nutrition service delivery. India has had an institutional set-up of a supervisory cadre since the inception of the nutrition program. However, the role played by supportive supervision at-scale and performance of CHWs has not been studied extensively. Studying the relationship between supportive supervision and performance of CHWs can provide future directions on whether investments in strengthening supervision could be a promising strategy to improve the nutrition service delivery, especially in low-resource settings.

This paper aims to fill this critical evidence gap using data from the Indian context to investigate the association between supportive supervision and CHW performance in a large-scale nutrition program using multi-level models. First, we assess whether the higher intensity of supportive supervision is associated with the better performance of CHWs. Second, we also examine whether the greater intensity of supportive supervision is associated with better performance through CHW’s knowledge. We define the *greater intensity* of supportive supervision as completing at least half the activities during supervisor’s monthly field visits to the Anganwadi Centre, conducting at least half the number of supportive supervision activities during supervisor–CHW monthly meetings, and delivering at least half the number of training topics on infant and young child feeding practices, and pregnancy and newborn care.

## Conceptual model

**Fig. 1:**
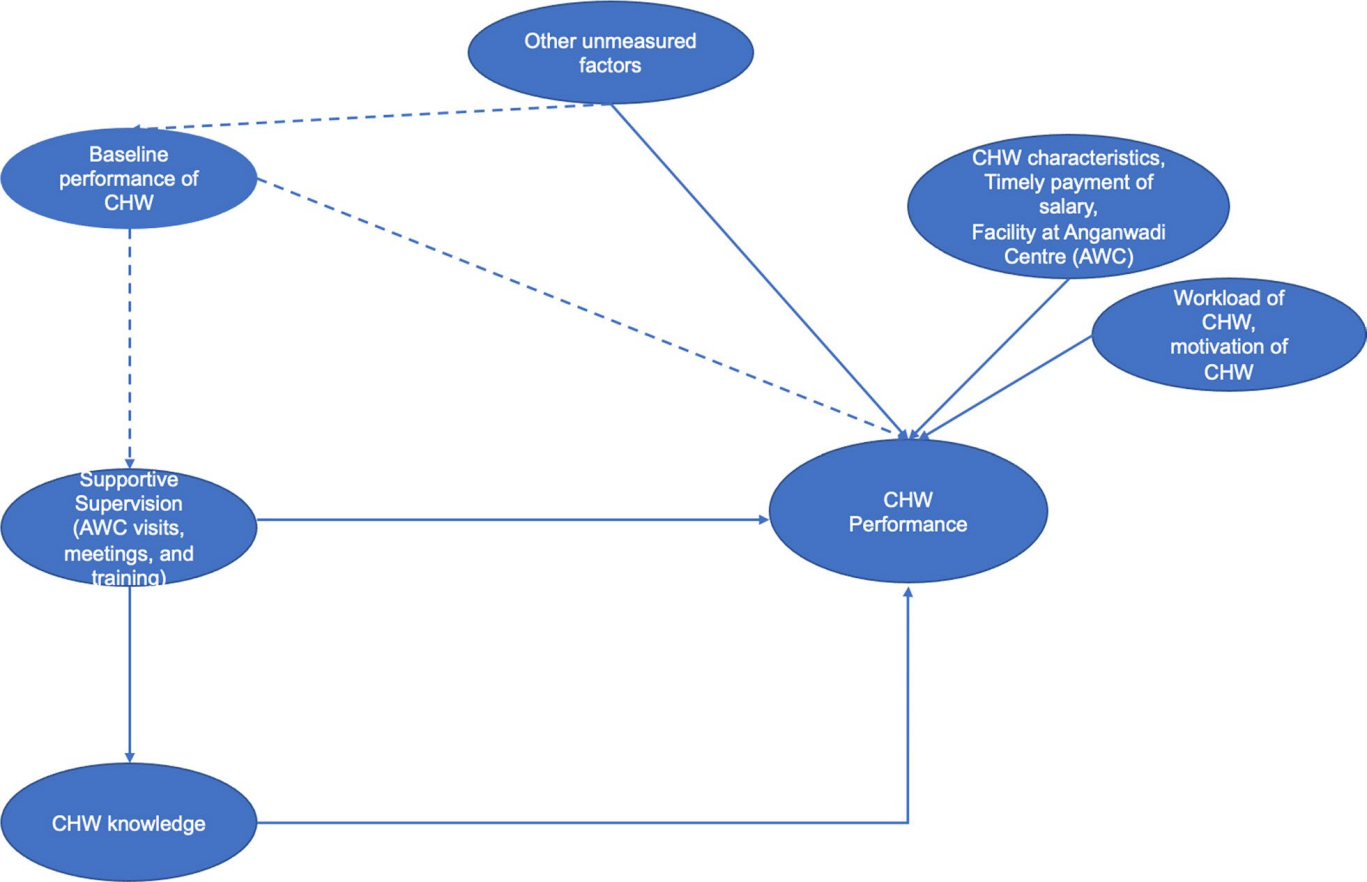
Conceptual model to study the association between supportive supervision and CHW performance

As shown in Fig. 1, we developed a conceptual model for the nutrition program's supervisory activities and existing supportive supervision and CHW performance frameworks [7, 27, 38]. Broadly, in the context of ICDS, supportive supervision includes monthly supervisor visits to the Anganwadi Centre and CHW–supervisor meetings to problem-solve, provide feedback, review records, and training sessions to improve service delivery of CHWs. Our model postulates pathways of how such a supportive supervision might affect performance directly through enforcing accountability on CHWs (which we could not measure in our surveys) or indirectly through increasing CHW knowledge through training.

Motivation is the degree to which an individual is willing to exert and maintain an effort towards an organization’s goals and has been recognized as a determinant of CHW performance [18]. We also consider CHW’s baseline performance as a confounder because (a) it can determine the intensity of supportive supervision she receives in the future, and (b) baseline performance may embed measured–unmeasured and time-invariant factors that drive CHW performance. For example, a highly motivated CHW will perform better. Our model also considers other CHW-level factors that can determine their performance but should not influence their supervisor’s behaviors. These factors considered in our model include job satisfaction (measured through timely receipt of salary), education, age, experience, infrastructure available in their AWC, and the population served by the CHW. Our model explicitly recognizes that unmeasured confounders will bias our results.

## Methods

### Study area

The data for this study come from an impact evaluation designed to test the effectiveness of a mHealth intervention for CHWs performance improvement. The study was conducted in 12 districts in the two north Indian states of Madhya Pradesh (MP) and Bihar between May 2017 and August 2019. More information on sample design and procedures are available in the impact evaluation protocol previously published [39].

### Participants and sampling

The endline survey of the impact evaluation collected data from 1344 CHWs located in 12 districts across MP and Bihar. However, because we control for the baseline performance of the CHW in our conceptual model, we used panel data of CHWs who were interviewed both at baseline and endline (*n* = 809). The final analysis set had 283 clusters and 809 CHWs with an average cluster size of 3 CHWs. Data were collected using structured computer-assisted personal interviews of CHWs by a trained enumerator. Indicators on AWC facility such as availability of water, toilet, electricity among others were collected via observation checklist by trained enumerators. All study participants provided verbal informed consent before data collection.

### Ethics

Impact evaluation study protocols were reviewed and approved by institutional review boards at the University of California, Berkeley (Ref. No. 2016-08-9092), and the India-based Suraksha Independent Ethics Committee (Protocol No. 2016-08-9092).

## Measurements

### Dependent variable

The dependent variable is constructed using an aided-recall by CHWs of the services they provided the month before the survey. We defined CHW performance based on whether CHWs reported delivering services as per the nutrition program guidelines. Performance is constructed as an additive score ranging from 0 to 5 based on the following five activities:

1. Conducting home visits to counsel women on pregnancy care and nutrition.
2. Conducting growth monitoring activity for children < 5 years.
3. Providing supplementary nutrition and take-home rations.
4. Organizing village health and nutrition day (like a health camp).
5. And providing pre-school education to children 3–6 years.

Government guidelines specify an average of 60 home visits each month, and if the CHW did at least half the number of home visits she was supposed to do, she received a score of 1, 0 otherwise. Similarly, for growth monitoring, children 0–11 months and 12–35 months are expected to be weighed monthly, and children 36–71 months are expected to be weighed quarterly. Accordingly, the indicator was constructed for weighing all children at appropriate intervals to be set to 1 if the CHW reported conducting growth monitoring activities and 0 otherwise. If the CHW reported providing age-appropriate food supplements, it was coded as a 1 and 0 otherwise. Pre-school education is to be provided daily to children 3–6 years. If the CHW responded that she provided it daily, it was coded as 1, otherwise 0. CHWs received a score of 1 if they organized at least 3 Village health and nutrition day in the three months preceding the survey, and 0 otherwise. An additive score ranging from 0 to 5 was calculated based on each service delivery score. We used the median as the cut-off to dichotomize the CHW performance additive score with those above the median to categorize *high/better performers* and those below the median as *low performers.*

### Independent variable

The nutrition program guidelines for supportive supervision include:

1. Monthly visits to the Anganwadi Centre to examine registers, discuss program-specific challenges, visually inspect AWC facilities, monitor CHW’s work on the field, accompany CHWs on home visits, and provide general guidance and help the CHWs navigate any challenges.
2. Monthly CHW-supervisor meetings during which supervisors review progress reports, discuss challenges, organize, and plan upcoming activities, discuss the performance of CHWs, and prioritize the list of beneficiaries who need further attention based on data.
3. Training the CHWs on health and nutrition topics, including infant, young child feeding practices, and pregnancy and newborn-related care.

Supportive supervision is a composite measure of both quantity and intensity of supervisory visits, monthly CHW-supervisor meetings, and training received by CHWs. The measure is calculated based on CHW’s report of (a) supervisory activities conducted by the supervisor during their recent visit to the AWC; (b) discussion of relevant topics during the monthly supervisor-CHW meetings, and (c) modules of training provided by the supervisor to the CHW. We assigned a score of 1 each if a supervisor had done at least half the supervisory activities during the visit to the AWC, discussed at least half the relevant topics during the monthly supervisor-CHW meetings, and completed at least half the number of training modules on infant young child feeding practices and pregnancy care. If a supervisor conducted all the above activities, they received a score of 1 and 0 otherwise. We defined supervisors who conducted all these activities as providing *greater intensity* of supportive supervision.

#### Covariates

We included the CHW characteristics of age (in years), education (years of schooling), caste (scheduled tribe/scheduled caste as marginalized caste with other backward classes and general caste as the reference group), the total population in CHW’s catchment area as a reflection of CHW workload, baseline performance, and timely receipt of salary by CHWs. Caste was used as one of the socio-economic variables using simplified categories per the government criteria based on the self-report by the CHWs (routinely used as a socio-economic indicator). The Indian caste system is a social stratification system through which ritual hierarchical and occupational status is ascribed to social groups and individuals. The current Indian government’s official caste classification system aggregates caste groups into four categories: Scheduled Caste (SC), Scheduled Tribe (ST), Other Backward Classes (OBC), and General Caste (GC). Scheduled Caste (SC) and Scheduled Tribe (ST) groupings together comprise the most marginalized in contemporary Indian society [40, 41]. The CHW motivation was measured using a survey question that examined the extent to which CHWs felt motivated to serve their community. enumerators were trained to record Anganwadi Centre (AWC) facilities and amenities by completing an AWC observation checklist. We used questions from the AWC observation checklist to develop an AWC facility index using a principal component analysis to combine the different facilities available at the AWC such as drinking water, functional toilet, electricity, whether AWC had a ‘pucca’ construction, sufficient indoor space for children, storage space to hold take-home rations, and salter scales meant to weigh children. For the CHW knowledge score, we used survey questions that tested CHW on knowledge related to complementary feeding, breastfeeding, newborn care, birth preparedness, and family planning. We calculated an additive score of the quiz questions ranging from a score of 0 to 47.

## Analytical approach

Unadjusted and adjusted hierarchical linear modeling (HLM) was done to examine the association between CHW performance and supervision. HLM is selected to account for correlation due to clustering and examine predictors at individual and cluster levels. The data are hierarchically arranged, with CHWs nested within clusters supervised by a supervisor-sector level.

We conducted a two-level analysis where CHWs (level 1) were nested within the supervision-sector (level 2); a supervisor typically manages 15–20 CHWs. We hypothesized that unobserved variables at the supervisor level could also be associated with CHW's performance in addition to her characteristics. We introduced district fixed effects to account for the fact that some districts that received the mHealth intervention (since the data come from an impact evaluation of this mHealth intervention) and control for other districts' characteristics. All analyses were done in Stata 15 [42]. We specified a mixed-effects model with a random intercept at the supervisor level and fixed effects at the district level. Logistic regression models were used to assess the probability of high performance of a CHW *i* in sector *j* in district *m* ${(Y}_{ijm }=1)$. Cluster-robust standard errors were used at the supervision-sector level.

The models build on one another in the following way: (1) a baseline model with only the primary independent variable (supervision) and the baseline performance of CHW because we hypothesized that their past performance could have influenced their recent supportive supervision and performance (2) the full model with the addition of CHW-level demographic variables including age, caste, education, experience, timely receipt of salary, CHW motivation, population served by CHW in their catchment area, and AWC facility index. The coefficients were transformed to odds ratios by exponentiating them for interpretation. We additionally conducted mediation analyses using the methodology developed by Baron and Kenny (1986) [43].

Then, to further assess whether the effect of supportive supervision on CHW performance is mediated by CHW knowledge, we first regressed CHW knowledge on supportive supervision using the same mixed-effects logistic regression models, followed by a second regression of CHW performance on supportive supervision. Finally, we regressed CHW performance on supportive supervision and CHW knowledge. CHW socio-demographic variables, motivation, average population in the catchment area, baseline performance, and baseline knowledge were controlled for each regression.

## Results

The demographics for the sample of 809 CHWs are described in Table 1. The average age of CHWs was 39 years, with an average of 15 years of experience as a CHW. On average, CHWs served a population size of 936 individuals in their catchment areas. An overwhelming majority of CHWs were currently married at the time of the survey, and 63% of CHWs had completed secondary education. About a third of CHWs belonged to the marginalized caste group (Scheduled Caste/Scheduled Tribe). Less than a quarter of CHWs reported receiving their salaries on time.

**Table 1:** Characteristics of CHWs and Anganwadi Centers (*n* = 809) at baseline in 283 sectors

Characteristics	*n*	% or mean (SD)
Mean age of CHW (in years)	809	39.0 ± 8.0
Average years of experience working as a CHW	809	15 ± 7
Average population in CHW’s catchment area	809	937 ± 301
CHW married at the time of the baseline survey	730	90%
**Caste of CHW**^a^		
General caste	219	27%
Other Backward Classes (OBC)	332	41%
Scheduled Caste/Scheduled Tribe	258	32%
**Highest education completed by CHW**		
Up to primary education (grades 1–8)	137	17%
Secondary education (grades 9–12)	513	63%
College education	159	20%
CHWs receiving timely salary in the 12 months preceding the survey	181	22%
CHWs who find their work to be motivating	752	93%
Anganwadi Center with ‘pucca’^b^ construction	587	73%
AWCs with drinking water	485	60%
AWCs with toilets	302	37%
AWCs with electricity	204	25%
AWCs with toddler scale for weighing children	241	30%
CHWs categorized as *better performers* at baseline	360	45%
CHWs who reported receiving *greater intensity* of supportive supervision at baseline	203	25%

^a^Scheduled Caste/Scheduled Tribe are considered marginalized caste groups

^b^Pucca refers to dwellings/buildings that are constructed to be permanent

Nearly three-fourths of the AWCs were situated in ‘pucca’ buildings (*pucca refers to dwellings/buildings that are constructed to be permanent*), and about 60% had drinking water within their premises. Only 37% of AWCs had functional toilets (toilets with water facilities), and about a quarter of them had electricity. Toddler scales for children were observed in about 30% of the AWCs. Among 809 CHWs, 45% of CHWs were categorized as *better performers* at baseline, providing age-appropriate services to their community as described earlier.

As shown in Table 2, the results indicate that those CHWs who receive a *greater intensity* of supportive supervision are more likely to be *better performers* than CHWs who receive lower intensity supportive supervision (OR 1.73, 95% CI 1.18, 2.54) adjusting for baseline performance of CHW. This association between supportive supervision and performance remains consistent even after controlling for CHW demographic characteristics, population served in their catchment area, motivation, AWC facility index, and timely receipt of CHW salary (OR 1.70, 95% CI 1.16, 2.49), signifying that supportive supervision is significantly associated with better performance of CHWs and not confounded by CHW characteristics and other factors we controlled for in Model 2. The full set of regression results is available in Additional file 1 (Table 3).

**Table 2:** Mixed-effects logistic regression results of supportive supervision on CHW performance (odds ratios and robust 95 percent confidence intervals)

	Model 1		Model 2	
	OR [*p*-value]	95% CI	OR [*p*-value]	95% CI
CHW who received greater intensity of supportive supervision	1.73 [0.005]	1.18,2.54	1.70 [0.007]	1.16,2.49
CHWs who were better performers at baseline	1.84 [0.000]	1.31,2.57	1.80 [0.001]	1.28,2.54
Sector-level variance (Level 2)	0.00		0.00	
Number of observations	809		809	

Fixed effect dummy for districts not shown in the table. Model 2 highlights the adjusted odds ratio after controlling for covariates such as CHW age, CHW education, CHW belonging to a marginalized caste, CHW experience, Anganwadi Centre facility index, timely receipt of salary, motivation of CHW, and the total population in CHW’s catchment area

**Table 3:** Coefficients and their significance in regression models establishing mediation for CHW performance, controlling for demographic characteristics, motivation, population served, baseline CHW performance and baseline knowledge (Odds ratios and robust 95% confidence intervals)

	CHW knowledge *X* → *M* $\left( {\hat{a}} \right)$		CHW performance *X* → *Y* $\left( {\hat{\tau }} \right)$		CHW Performance ({*X*, *M*} → *Y*) $\left( {\hat{\tau }^{\prime}} \right)$	
	OR [*p*-value]	95% CI	OR [*p*-value]	95% CI	OR [*p*-value]	95% CI
Supportive supervision	4.65 [*p* = 0.000]	3.18,6.79	1.70 [*p* = 0.007]	1.16,2.49	1.40 [*p* = 0.092]	0.95,2.07
CHW knowledge (*M*, *β*)					1.82 [*p* = 0.000]	1.31,2.51

*M* refers to mediator (CHW knowledge); *Y* refers to CHW performance

### Mediation effect

As shown in Table 3, greater intensity of supportive supervision was significantly associated with both CHW knowledge (*α* = 4.65, *p* = 0.000) and CHW performance (*τ* = 1.70, *p* = 0.007) when regressed separately. When CHW knowledge and intensity of supportive supervision were introduced in the model to estimate their effect on CHW performance, the effect of greater intensity supportive supervision on CHW performance (*τ*′ = 1.40, *p* = 0.092) and the effect of CHW knowledge on CHW performance (*β* = 1.82, *p* = 0.000) were statistically significant at conventional levels, suggesting that CHW knowledge mediates the path of supportive supervision to CHW performance. However, it is not a perfect mediator.

## Discussion

Our analysis confirms that greater intensity of supportive supervision is associated with better CHW performance after controlling for time-invariant confounders affecting CHW performance and other covariates that can affect her performance. The results also mean that CHWs who perform well at baseline are more likely to continue performing well, controlling for supportive supervision. Our mediation analysis suggests that supportive supervision indirectly increases CHW knowledge and directly through the accountability pathway, as we hypothesized in the conceptual model.

This study is consistent with some extant evidence highlighting the importance of supportive supervision in CHW programs and their contribution to improved CHW performance. Aftab and colleagues in Pakistan found some evidence indicating that enhanced supportive supervision improved CHWs’ ability to assess dehydration and classify diarrhea correctly [44]. A dated study from rural India also found that regular supportive supervision augmented the performance of CHWs in home-based neonatal care and strengthened their knowledge of basic tasks such as taking temperature, weighing of neonates, and providing breastfeeding education [45]. However, some previously published studies did not observe any impact of supervision on CHW services and health outcomes [18, 28, 46].

Some scholars have argued that inconsistent evidence of impact of supportive supervision stems from a limited understanding of what constitutes supportive supervision and how the different components of supportive supervision influence CHW performance in diverse contexts and settings [21]. Even when the evidence exists in different contexts, it is often not sufficiently granular to recommend which supportive supervision strategies are most effective [20]. Depending on the context and whether supervision is scaled-up or piloted as an intervention, there is a need to better understand how supportive supervision works and in what contexts. Our study explores this in a limited way, but we cannot disentangle which approaches used by the supervisors work better than others.

Our findings indicate that strengthening the existing mechanism of supportive supervision of CHWs in India’s Integrated Child Services Development (ICDS) can result in better performance of CHWs. Our findings also suggest that supportive supervisory paradigms that focus less on inspection and record reviews and focus more on strengthening the skills and knowledge of CHWs on a regular and ongoing basis hold promise.

Overall, this evidence is timely with India’s POSHAN Abhiyaan (National Nutrition Mission) launch. There is a recent impetus on supportive supervision checklists for Anganwadi Center monitoring visits conducted by supervisors. This checklist enables the supervisors to focus more on understanding the status of undernutrition and service delivery at the AWC and support the CHWs in delivering services more effectively. The study from India discussed earlier found that checklists allowed for better two-way communication between supervisors and CHWs [26]. In global settings, several studies have reported using either a supervisory checklist or guidelines to assist supervisors in conducting supervision though the effectiveness of such checklists has not been evaluated [18, 47–50]. We hope that future studies can document the extent to which these checklists’ use further improves supervisory support to CHWs.

Our study contains some limitations that deserve consideration. The use of observational data reduces our ability to make any causal inference due to unmeasured confounding, even though we included several covariates to reduce confounding, and the results of Model 1 and Model 2 (in Table 2) are highly comparable. We have studied knowledge as a mediator, but there could be other unmeasured mediators. Our measurements are based on self-reports by CHWs and subjective measurements could be prone to recall and social desirability bias. There is no agreed metric for measuring CHW performance or supportive supervision [38], so we created our indices and dichotomized them. Future research should develop validated measures and scales that can capture and quantify CHW performance and supervision. Since the communities were sampled for the larger impact evaluation study using propensity score matching methods, this sample should not be considered a statistically representative sample of the CHW population in these states. Lastly, we acknowledge as a study limitation the inability to thoroughly examine the impact of caste on the supervisor–CHW relationship and the CHW–beneficiary relationship. Future studies should consider examining the impact of caste on CHW performance.

## Conclusion

Our study finds that greater intensity of supportive supervision that includes sufficient monitoring visits by the supervisor, CHW-supervisor meetings, and training is associated with higher CHW performance even when controlling for CHW heterogeneity. Higher intensity supportive supervision improves CHW performance in two ways: directly through accountability measures and indirectly through increasing the knowledge and skills of CHWs, enabling them to serve their communities better. Leveraging an extant institutional mechanism such as supportive supervision within the Integrated Child Services Development program can be a valuable strategy to improve the performance of CHWs. Efforts to support the performance of CHWs through strengthening supportive supervision could be significant in enhancing service delivery to reach millions of mothers and children in the country and potentially improve infant and young child feeding practices and nutritional outcomes.

## Supplementary Information

**Additional file 1: Table S3.** Full regression results of supportive supervision on CHW performance (odds ratios and robust 95 percent confidence intervals)

## Data Availability

The datasets analyzed in the current study are not publicly available given that the research team has not published results from the main impact evaluation study but are available from the corresponding author on request.
