# Supplementary material for: Association between supportive supervision and performance of community health workers in India: a longitudinal multi-level analysis
Source: Hum Resour Health. 2021 Nov 27;19:145. doi: 10.1186/s12960-021-00689-7 (PMC8627081; doi:10.1186/s12960-021-00689-7)
Supplement: Supplementary file 1 — Additional file 1: Table S3. Full regression results of supportive supervision on CHW performance (odds ratios and robust 95 percent confidence intervals) [file 12960_2021_689_MOESM1_ESM.docx]

Supplementary Table

*Table 3: Full regression results of supportive supervision on CHW performance (Odds ratios and robust 95 per cent confidence intervals)*

|  | **Model 1** | | **Model 2** | |
| --- | --- | --- | --- | --- |
|  | OR  [p-value] | 95% CI | OR  [p-value] | 95% CI |
| **Higher quality supportive supervision** | 1.73  [0.005] | 1.18,2.54 | 1.70  [0.007] | 1.16,2.49 |
| **Past performance of CHW** | 1.84  [0.000] | 1.31,2.57 | 1.80  [0.001] | 1.28,2.54 |
| **CHW age** |  |  | 1.00  [0.999] | 0.97,1.03 |
| **CHW education** |  |  | 1.09  [0.003] | 1.03,1.16 |
| **CHW belongs to marginalized caste** |  |  | 1.00  [0.992] | 0.64,1.54 |
| **CHW experience** |  |  | 1.02  [0.133] | 0.99,1.05 |
| **AWC facility index** |  |  | 1.02  [0.922] | 0.70,1.48 |
| **Regularity of CHW salary** |  |  | 1.36  [0.169] | 0.88,2.11 |
| **Motivation of CHW** |  |  | 1.58  [0.179] | 0.81,3.09 |
| **Total population in AWC catchment area** |  |  | 1.00  [0.760] | 1.00,1.00 |
| **Sector-level variance** | 0.00 |  | 0.00 |  |
| **Number of Observations** | 809 |  | 809 |  |
